# Investigating barriers to adherence to antimalarial prescribing guidelines in public healthcare facilities in Arba Minch, South Ethiopia: A qualitative study

**DOI:** 10.1371/journal.pone.0337326

**Published:** 2025-11-26

**Authors:** Abate Atimut Dereje, Dereje Geleta, Tadesse Menjetta, Abinet Takele, Susana Vaz Nery, Techalew Shimelis

**Affiliations:** 1 College of Medicine and Health Sciences, Wolkite University, Wolkite, Ethiopia; 2 College of Medicine and Health Sciences, Hawassa University, Hawassa, Ethiopia; 3 College of Medicine and Health Sciences, Arba Minch University, Arba Minch, Ethiopia; 4 Kirby Institute, University of New South Wales, Sydney, Australia; Sunu Sante Consulting, SENEGAL

## Abstract

**Background:**

Early diagnosis and prompt treatment of malaria cases are a crucial component of curative and preventive interventions. There have been reports of healthcare workers overprescribing antimalarial agents against guidelines, but the barriers they face in adhering to the guidelines are not well studied. This study aimed to investigate barriers to adherence to guidelines in prescribing antimalarial drugs in public healthcare facilities in Arba Minch, South Ethiopia.

**Method:**

A cross-sectional descriptive exploratory qualitative method was employed. We included ten participants from public healthcare facilities, including health centres, a hospital, a city health office, and a zonal health bureau. A key informant interview technique was used to collect data. All interviews were audio-recorded, transcribed, and analyzed. Data analysis was performed using ATLAS.ti, version 7.5 software. The results were presented thematically and narrated to support the main themes.

**Results:**

Public healthcare facilities primarily used blood smear microscopy to test all malaria-suspected patients. However, in cases of microscopy service interruptions or when confirming negative results, rapid diagnostic tests (RDTs) were employed in some facilities. Limited availability of microscopes and reagents, and electric power interruptions hindered reliable microscopy services. Drug stock-outs, patient expectations for antimalarial drugs, self-treatment, and delayed care-seeking are barriers to adherence to malaria treatment guidelines. The main reason for non-adherence to withholding antimalarial drugs after negative tests was greater trust in clinical findings over laboratory results. Confidence in experience contributed to trust in clinical judgment, while perceived inexperience and negligence, inconsistent RDT and microscopy results, and poor-quality control assessment results undermined trust in laboratories. Despite supporting the guidelines, study participants emphasized the need for flexibility to allow empirical treatment and highlighted the lack of training and mentoring for healthcare workers.

**Conclusion:**

To improve adherence to malaria treatment guidelines, it is essential to ensure consistent lab operations, enhance quality assurance, maintain effective communication between lab personnel and prescribers, and provide healthcare and patient education. Implementing training and mentoring programs and promoting evidence-based practices are also crucial.

## Introduction

Malaria remains a serious global health problem, particularly in the World Health Organization (WHO) African Region, which accounts for 94% of malaria cases (233 million) and 95% of deaths (580,000) in 2022. Children under five years of age account for about 78% of all malaria deaths in the region. From 2019 to 2022, the number of malaria cases in this region rose from 218 million to 233 million [1].

In Ethiopia, about 68% of the population is at risk of malaria, with transmission being seasonal and unstable [2]. *Plasmodium falciparum* and *P. vivax* cause 65% and 35% of cases, respectively [3]. Malaria cases decreased significantly from 5.5 million in 2010 to less than 1 million in 2019, while deaths dropped from 3,000 in 2010 to just 212 in 2021 [4]. However, disruptions from the coronavirus disease pandemic, conflict, and displacement led to a 32.5% increase in confirmed cases between 2021 and 2022, reaching 1.5 million. In 2023, cases surged by 150% and 120% compared to the same periods in 2021 and 2022, respectively [5].

Accurate diagnosis and prompt treatment are essential components of malaria control [6]. Blood smear microscopy is regarded as the gold standard for diagnosis [7] and is used in Ethiopian healthcare facilities, while rapid diagnostic tests (RDTs) are recommended for community health posts and remote areas where microscopy is unavailable. Guidelines emphasize the need for parasitological (microscopy or RDT) confirmation of malaria before prescribing antimalarial agents and recommend follow-up of negative test results without administering antimalarial treatment. This aims to minimize antimalarial drug resistance and identify febrile patients without malaria for appropriate diagnosis and treatment [8,9].

In Ethiopia, adherence to guidelines for negative test results differs by healthcare facility type [10–14]. For example, in Hawassa, healthcare professionals at lower-level public healthcare facilities (health centres) adhered to negative test results 100% of the time [13], while those at a higher-level facility (tertiary hospital) adhered to around 93% of the time [13,14]. In Arba Minch, adherence was also high in the general hospital (98.6%) and health centres (100%) [11]. By contrast, studies from private clinics reported adherence rates of 4.2% [12] and 95.9% [10]. However, these quantitative studies may not fully reflect the prescribing practices of healthcare workers in the study sites, as they are based on a small number of professionals working in the clinics during the study period. Therefore, qualitative studies are essential to provide a broader understanding of adherence to the guidelines. This study aimed to investigate barriers to adherence to antimalarial prescribing guidelines among healthcare professionals in public healthcare facilities.

## Materials and methods

### Study design and setting

A cross-sectional descriptive exploratory qualitative study design was conducted in December 6 to 11, 2023, in public healthcare facilities in Arba Minch City, located about 500 km south of Ethiopia’s capital, Addis Ababa. Arba Minch, situated in the Gamo Zone of the South Ethiopia Region, had a projected population of 201,049 in 2022, covering 32.97 km^2^ with a population density of 6,098 people per km^2^ and an annual growth rate of 6.8%. The city is surrounded by water bodies, including Lake Abaya, Lake Chamo, and the Kulfo River, which provide breeding sites for mosquitoes and increase malaria transmission. Arba Minch has two public hospitals, one private hospital, two health centres, and 50 private clinics, which are crucial for delivering curative and preventive malaria services.

### Study participants

Participants were purposively selected based on their professional roles, expertise, and relevance to the study topic to ensure the collection of in-depth insights. Their active engagement in malaria diagnosis, treatment, supervision, monitoring, training, and overall program coordination at various levels of the health system positioned them as key informants with valuable experience and perspectives essential to achieving the study objectives. This included the public health emergency management (PHEM) focal persons, malaria focal persons, and physicians and health officers from health facilities, as well as from district and zonal health departments. A total of ten respondents were interviewed. The sample size was determined considering available time and budget for the study. Importantly, information saturation was also considered to ensure that sufficient and relevant data had been collected.

### Data collection techniques

Data were collected through key informant interviews (KII) using interview guides. KII were preferred due to the nature of the study participants, who held expert-level knowledge and decision-making power. They served in leadership, technical, and administrative capacities. The interviews aimed to gather information on barriers related to malaria diagnosis and treatment, adherence to antimalarial drug prescribing guidelines, reasons for non-adherence, and recommendations for overcoming these challenges. To minimize bias, including potential preconceptions, we recruited an experienced data collector to facilitate the interviews with the first author, thereby reducing the influence of the researchers’ backgrounds on interview interactions. We also conducted debriefing sessions among the research team, guided by the interview notes provided by the data collector. The date and place of each interview were arranged through consultation with the participant beforehand. All interviews were audio-recorded. The duration of the interviews ranged from 30 to 50 minutes. Interview notes taken during the sessions were used to supplement the audio recordings to enhance the quality of the collected information. The interviews were facilitated by two experienced qualitative researchers. The interview guide was pre-tested to assess its clarity, appropriateness, structure, flow, and the effectiveness of probing for depth before the study commenced.

### Data management and analysis

All key informant interviews were transcribed verbatim and then translated from Amharic (the local language) to English. The first and second authors independently assigned codes to each interview. Initially, they had read through all transcripts to become familiar with the data. Guided by the second author, codes were generated inductively. To enhance analytic rigor, the first author refined the generated codes to reflect recurring patterns. The coded data were then organized into categories, and themes were developed by identifying patterns across categories that aligned with the research objectives. Peer debriefing with the research team was conducted throughout the analysis to ensure consistency. Data analysis was performed using ATLAS.ti, version 7.5 (ATLAS.ti Scientific Software Development GmbH, Berlin, Germany). Thematic analysis was conducted, and themes were developed to elaborate on and narrate the main findings. Results were presented using themes and narrative descriptions**.**

### Ethical approval

This study was approved by the Institutional Review Board of Hawassa University’s College of Medicine and Health Sciences (Ref No: IRB/198/15, IRB/AM001/16). Participation was fully voluntary, and written informed consent was obtained from participants. All data gathered from respondents was kept confidential.

## Results

### Participants’ profile

A total of 10 participants were included in the study. From the Zonal Health Bureau, two participants were included: a PHEM focal person and a malaria focal person. From the City Health Office, a malaria focal person participated. At the hospital level, three participants were interviewed: a malaria focal person, a pediatrician, and a general practitioner. From the health centres, four participants took part: a PHEM focal person, a malaria focal person, and two health officers working in the under-five outpatient department (OPD). Nine participants were male and one was female. Participants held educational qualifications such as a Bachelor of Science, Master of Public Health, and Doctor of Medicine degrees. They represented diverse professional fields such as Nursing, Public Health, Medicine, Pediatrics and Child Health, and Medical Laboratory Science, and had between 2 and 28 years of experience in the health sector.

### Key themes

We developed four key themes: barriers to malaria diagnosis and treatment, factors contributing to non-adherence in prescribing antimalarial drugs, availability of guidelines and training, and healthcare workers’ attitudes toward guidelines. Each key theme also includes relevant sub-domains.

#### Theme 1: Malaria diagnosis and treatment barriers.

**Malaria diagnosis and barriers:** Most of the study participants indicated that all patients suspected of having malaria were tested using blood smear microscopy at healthcare facilities and RDTs at health posts, following national guidelines. RDTs were also used at some healthcare facilities to confirm negative microscopy results or when microscopy services were interrupted.

Respondents stated:

*“After assessing signs and symptoms, if we suspect malaria, we send the patient to the laboratory for diagnosis”* (Public Health Officer, 20 years’ experience).

*“The diagnosis is done using blood smear microscopy”* (Nurse, 10 years’ experience).

*“We only use RDT to confirm negative microscopy results; otherwise, the guidelines do not recommend it in healthcare facilities. We do this to increase the positivity rate*” (Pediatrician, 5 years’ experience).

*“RDT is used at the health post level… for us [health centre], it might be against the guidelines. We used to use RDTs during electric power interruptions”* (Public Health Officer, 5 years’ experience).

Healthcare workers considered RDT results more reliable than microscopy and used RDTs as confirmatory tests.

Barriers to performing malaria microscopy included shortages of reagents, limited availability of microscopes, and power outages:

*“Due to electric power interruptions and reagent shortages, some facilities do not perform microscopy, especially in remote areas”* (Public Health Officer, 17 years’ experience).

*“There is a shortage of microscopes. Patients must wait a long time for laboratory results, which can have clinical implications for children. Repeat testing impacts lab personnel and exacerbates the shortage of manpower”* (Nurse, 10 years’ experience).

*“RDT is only allowed for health posts. When we had no electric power, we sent patients to a nearby healthcare facility”* (Public Health Officer, 5 years’ experience).

Some of the participants noted that repetitive follow-up laboratory testing increased workload and made patients or caregivers uncomfortable due to the additional costs.

“*A nurse may follow up with five or more patients. The severity of symptoms is another issue. Hence, testing a single patient three or four times a day is burdensome”* (Medical Doctor, 2 years’ experience).

Managing the discrepancy between laboratory results and patient expectations was also challenging, as patients or caregivers in the endemic areas, familiar with malaria symptoms, often felt uneasy receiving negative test results at public healthcare facilities. Thus, patients preferred private clinics where they might receive positive test results and antimalarial medication.

“*The challenge is that most of the time, microscopy yields negative results for malaria. When patients have symptoms, they might not be satisfied with the results, leading them to visit private clinics where their malaria test results are reported as positive”* (Public Health Officer, 20 years’ experience).

*“Most of the time, blood smear microscopy yields negative results for malaria. I think it is individual-dependent. I do not know the reason, but the results are often negative for malaria. The RDT positivity rate is very high, although there is a tendency to show false positives”* (Pediatrician, 5 years’ experience).

In addition to the aforementioned diagnostic challenges, most of the participants cited issues such as a lack of experience and negligence among laboratory technologists, inconsistent results between RDT and microscopy, and poor-quality control assessment results in laboratories. These factors contributed to a lack of trust in laboratory results among healthcare workers and patients.

*“Lab technologists have skill gaps. Sometimes, they do not report the results accurately. There are instances where a positive malaria test result is reported as negative. Explaining why a positive result is reported as negative is challenging”* (Public Health Officer, 15 years’ experience).

“*Occasionally, a less experienced lab technologist might diagnose a patient as negative for malaria while a more experienced technologist would diagnose the same patient as positive”* (Public Health Officer, 20 years’ experience).

*“There might be misidentification of the species. For example, after a patient is diagnosed with P. vivax and takes medication for three days without relief, the patient might return and be diagnosed with P. falciparum in the next lab test”* (Medical Laboratory Technologist, 7 years’ experience).

*“We conduct quality control. The regional lab performs quality control quarterly. There was a significant gap (about 85%) where slides were read incorrectly at one of the health centres”* (Public Health Officer, 28 years’ experience).

*“In most meetings, it is noted that laboratory technologists have a skill gap in identifying Plasmodium species”* (Public Health Officer, 5 years’ experience).

“*Sometimes, lab personnel may rush to declare negative results after examining only a few microscopic fields. The community also identified individuals in the laboratory who can identify malaria and those who cannot”* (Public Health Officer, 28 years’ experience).

**Malaria treatment and barriers:** Malaria was treated in the study setting using Coartem (artemether-lumefantrine), chloroquine, and primaquine, depending on the species of *Plasmodium*. The study participants indicated that while it was against the guidelines, children with severe illnesses were treated with intravenous artesunate. Healthcare workers knew the appropriate drugs for each *Plasmodium* species.

*“We prescribe chloroquine and primaquine for P. vivax and Coartem for P. falciparum. If the symptoms persist, we will follow up with the patient”* (Public Health Officer, 20 years’ experience).

Most of the healthcare workers reported that the main challenge with treatment was antimalarial drug stock-outs:

“*When we run out of antimalarial drugs, we advise the patients to buy them from a private facility. This option is often prohibitively expensive for many of our clients”* (Nurse, 6 years’ experience).

In addition, self-treatment in urban communities and delayed treatment-seeking in rural areas presented challenges to malaria treatment.

*“Patients from urban areas typically visit healthcare facilities for antimalarial drug prescriptions, often after first attempting home treatment. Patients from rural areas usually seek care when the illness has become severe”* (Nurse, 10 years’ experience).

*“Most urban residents rely on self-treatment with antimalarial drugs, and come to see us when their illness fails to improve”* (Nurse, 10 years’ experience).

“*The community is familiar with malaria symptoms and has access to alternative sources of treatment. If they are not given drugs at the health centre, they can obtain them outside at private facilities. This is common practice in malarious areas”* (Public Health Officer, 15 years’ experience).

#### Theme 2: Factors for non-adherence in prescribing antimalarial drugs.

Most of the study participants highlighted instances where healthcare workers deviated from guidelines by prescribing antimalarial agents to patients with negative test results. Factors contributing to empirical treatment included the high endemicity of malaria and the presence of characteristic malaria symptoms despite repeated negative test results.

*“The prevalence of malaria is high in this area. A patient presenting with a high fever from an unknown cause is treated for malaria clinically”* (Medical Doctor, 2 years’ experience).

“*When patients are repeatedly tested and are found negative for malaria, but the symptoms continue to support malaria, the professionals become confident that the diagnosis is malaria”* (Nurse, 6 years’ experience).

Additionally, the positive outcomes associated with empirical treatment and the potential negative consequences of withholding antimalarial drugs from patients with negative test results influenced adherence.

*“When we observe basic symptoms of malaria such as anemia, splenomegaly, intermittent fever,* *and the like, we treat empirically and often see recovery. We do this because the malaria negativity rate is very high”* (Pediatrician, 5 years’ experience).

*“Most of the time, the result is negative. We repeat it at least three times. It might still become malaria-negative. When we treat using only clinical diagnosis, we see changes”* (Pediatrician, 5 years’ experience).

*“Although malaria-related deaths are not occurring, patients might experience complications if we strictly adhere to the guidelines and do not provide treatment for negative cases”* (Public Health Officer, 5 years’ experience).

Healthcare professionals prioritized clinical findings and their own experiences over laboratory results when there was a discrepancy, which led to the prescription of antimalarial agents despite negative test results.

Other factors influencing adherence to guidelines included a lack of training on updated guidelines and the turnover of trained healthcare workers, as experienced professionals leave the workplace.

*“Due to staff turnover, new personnel rely on their previous experience since they are not trained for it”* (Public Health Officer, 17 years’ experience).

*“We found them prescribing artesunate improperly. We also observed this gap among senior physicians…. The underlying reason for non-adherence is a lack of mentorship”* (Public Health Officer, 17 years’ experience).

Pharmacists were mandated to dispense antimalarial drugs only with a prescription that included laboratory results. Hospital pharmacists generally adhered strictly to this directive, refusing to dispense the drugs without the required lab results and even demanding confirmation for positive referrals.

*“Unless a positive malaria result from the laboratory is attached, pharmacists will not dispense antimalarial drugs in the hospital”* (Medical Doctor, 2 years’ experience).

*“They do not even give antimalarial drugs for referral cases based on laboratory results from other healthcare facilities. It must be a positive result from this hospital”* (Medical Doctor, 2 years’ experience).

Physicians’ heavy reliance on clinical findings over lab results led them to bypass pharmacists by persuading lab technologists to alter negative results to positive, undermining an adherence control mechanism.

Participants stated:

*“Presently, clinicians sometimes come to us, describing the symptoms and suggesting that it would be better to provide a prescription. Then, we [laboratory technologists] may write a positive result; even if the actual test result is negative” (Medical Laboratory Technologist, 7 years’ experience).*

*“Sometimes, the Plasmodium parasite may be at a stage that is difficult to detect. Thus, we use RDTs for confirmation. If the result remains negative, we report it as such. However, when clinicians express a high suspicion of malaria, we may alter the result to positive so that the patient can receive medication from the pharmacy”* (Medical Laboratory Technologist, 7 years’ experience).

*“Pharmacists require a positive test result from the laboratory to dispense antimalarial agents. For patients exhibiting malaria signs and symptoms but with negative test results, especially those from distant areas who cannot return for follow-up testing within 6 hours, we may alter the result from negative to positive. We also change results for patients who have been tested two or three times with negative results while still showing symptoms”* (Medical Laboratory Technologist, 7 years’ experience).

*“Pharmacists do not dispense antimalarial drugs based on negative test results. However, as mentioned earlier, we prescribe these drugs for malaria-negative test results in critical cases, following discussions with laboratory technologists”* (Medical Doctor, 2 years’ experience).

Most of the participants also noted that the non-adherence practices contributed to early antimalarial drug stock-outs, misuse of drugs and misallocation of resources.

*“Those medicines [prescribed for negative cases] are available on the market, which can lead to misuse and affect overall government resource allocation*” (Public Health Officer, 17 years’ experience).

*“This [prescribing for negative cases] affects the antimalarial drug supply, relative to the positive malaria reports”* (Public Health Officer, 28 years’ experience).

*“Sometimes, the medicines requested [prescribed for negative cases] do not align with the identified disease. It was found that drugs worth 40 million birr were requested improperly, relative to the national disease prevalence rate”* (Public Health Officer, 17 years’ experience).

#### Theme 3: Availability of guidelines and training.

Various guidelines and manuals were available at healthcare facilities to assist healthcare workers in diagnosing and managing illnesses, including malaria, in adults and children. The integrated management of neonatal and childhood illness (IMNCI) was widely used at health centres for managing children under five years old. The zonal health department distributed guidelines and manuals to both private and public healthcare facilities.

Respondents noted:

“*Standard guidelines were provided to each health centre and malaria focal persons were trained on the guidelines. Manuals were given to each district following the training”* (Public Health Officer, 17 years’ experience).

“*We have under-five and adult management guidelines. We use IMNCI guidelines for under-five children”* (Public Health Officer, 5 years’ experience).

Training on malaria diagnosis and treatment guidelines was provided to district malaria focal personnel, PHEM, and disease prevention officers. However, only physicians at health centres received training on these guidelines. Limited access to training opportunities, a lack of resources, and political instability in the region were identified as potential barriers to training all healthcare workers across all facilities. Despite the training limitations, most participants noted that guidelines were available in their healthcare facilities. The guidelines were distributed in both print and digital formats to address the shortage of resources.

“*Last year (2023), for instance, the district PHEM officer, malaria officer, disease prevention officer, and five individuals from each of the 14 districts were trained and certified. The malaria focal person was trained at the facility level”* (Public Health Officer, 17 years’ experience).

“*The guidelines are distributed in both printed and digital formats to public and private sectors. For five private clinics, we provided digital copies for treating malaria using RDT and Coartum after signing the necessary memo”* (Public Health Officer, 28 years’ experience).

Most of the participants reported insufficient training on the updated guidelines, with both officers and healthcare workers highlighting the need for additional training.

“*Since the introduction of the new guidelines, there has been no training provided; only the guidelines were sent. We laminated and distributed the guidelines to every healthcare facility*” (Public Health Officer, 28 years’ experience).

“*I suggest providing training for all healthcare professionals. The responsibility currently falls on those who have been trained, which is insufficient. Everyone should be aware and involved. At times, there is staff turnover, which creates a problem”* (Medical Doctor, 2 years’ experience).

#### Theme 4: Healthcare workers’ attitude towards guidelines.

Almost all participants supported guidelines that emphasize assessing for non-malaria infections in patients with negative malaria test results, rather than treating based solely on the area’s malaria endemicity.

The participants stated that,

*“What I reject is the idea that, simply because the area is malaria-endemic, treatment should not be determined only by clinical diagnosis. It makes us overlook other diseases”* (Medical Doctor, 2 years’ experience).

However, some of the participants suggested that the guidelines should allow flexibility for empirical treatment not only for severe cases but also for other patients. They stressed the importance of considering clinical judgments even when test results are negative, due to a lack of trust in laboratory results and the burden of repetitive laboratory tests and case follow-ups.

The study participants stated:

*“I observed that 80% of the signs and symptoms were consistent with malaria, yet the laboratory result was negative. There could be an error in the microscopy or RDT”* (Public Health Officer, 20 years’ experience).

*“It is problematic to rely solely on the test results. There should be an option to prescribe medication based on clinical signs and symptoms”* (Public Health Officer, 20 years’ experience).

“*Laboratory quality, the quality of RDT devices, and the experience of laboratory technologists can influence the accuracy of test results. Therefore, there should be room to rely on clinical signs and symptoms as well”* (Public Health Officer, 5 years’ experience).

“*I support the guidelines, but there should be flexibility to clinically diagnose severe cases. Repetitive lab testing for negative results every hour can create a burden”* (Pediatrician, 5 years’ experience).

Mainly, senior healthcare workers resisted ignoring the established practices of empirical treatment because they did not trust that strict adherence to guidelines guarantees desirable clinical outcomes.

*“Physicians may resist adhering to guidelines and sometimes base treatment decisions solely on clinical signs and symptoms”* (Public Health Officer,17 years’ experience).

**Fig 1:**
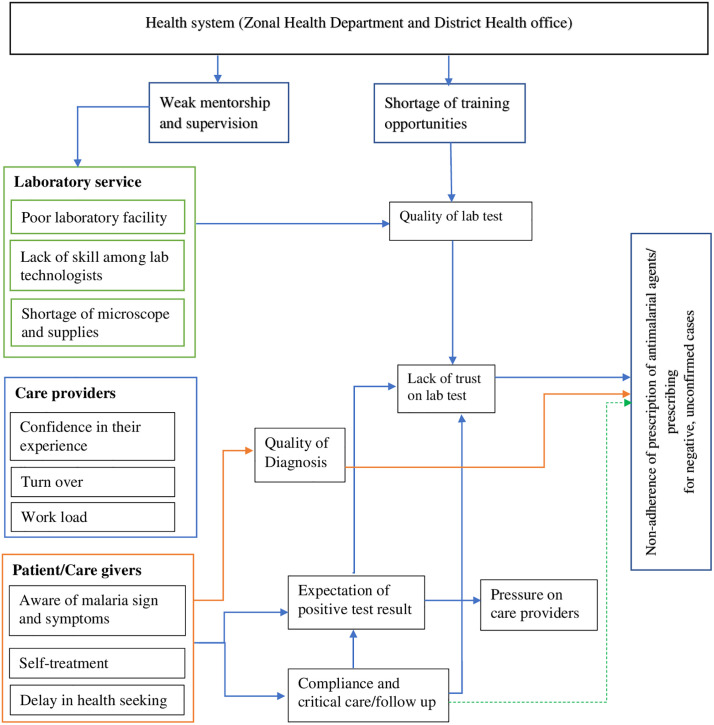
Complexity of barriers driving non-adherence to guidelines when prescribing antimalarial agents, Arba Minch, 2023.

In summary, non-adherence to antimalarial prescribing guidelines was multifaceted. This study identified key barriers at multiple levels, including the health system, laboratory services, healthcare providers, and patients and caregivers (Table 1). Furthermore, the interactions among these barriers were complex and interdependent (Fig 1).

**Table 1:** Domains and sub-domains of factors driving non-adherence to antimalarial prescribing guidelines among healthcare workers, Arba Minch, 2023.

Domain	Sub-domains
Health system (zonal health department and district health office)	Weak mentorship and supportive supervision
	Lack of training opportunities
Laboratory service	Poor laboratory quality control assessment feedback
	Lack of skill among laboratory technologists
	Shortage of microscopes and reagents
Healthcare providers	Confidence in their experience
	Trained staff turnover
	Increased workload
Patients and caregivers	Awareness of symptoms
	Self-treatment
	Delayed healthcare-seeking

## Discussion

This study explored the barriers to antimalarial prescribing guidelines in public healthcare facilities, focusing on themes such as malaria diagnosis and treatment challenges, reasons for non-adherence, availability of guidelines and training, and attitudes towards adherence. Participants noted that all patients suspected of having malaria were tested using blood smear microscopy at healthcare facilities. However, the limited availability of microscopes and reagents, along with power outages, hindered uninterrupted malaria microscopy services. Drug stock-outs, self-treatment and delayed care-seeking also pose significant challenges to malaria treatment. The main reason for non-adherence to withholding antimalarial drugs after negative tests was trust in clinical findings over laboratory results. Participants emphasized the need for flexibility within the guidelines and highlighted the lack of training and mentoring for healthcare workers.

Healthcare workers’ heavy reliance on clinical findings and subsequent non-adherence to guidelines mainly stemmed from a lack of trust in microscopy results, exacerbated by inconsistent microscopy services. Contributing factors included the perceived inexperience and negligence of laboratory technologists, inconsistent results between RDTs and microscopy, and poor-quality control assessment feedback. These findings align with earlier studies in Africa [15–17]. RDTs, intended for use at community health posts, helped mitigate microscopy service interruptions due to power outages and confirm negative microscopy results when suspicion remained high, aiding clinical decision-making. However, RDTs cannot help estimate parasite density or monitor treatment response, highlighting the need for establishing reliable microscopy services [18,19]. This requires alternate power sources and a steady supply of reagents. Strengthening quality assurance programs to address laboratory personnel skill concerns, ensuring the release of quality results, and fostering good communication with healthcare workers are essential to building trust and understanding of laboratory results. Further, integrating diagnostic tools to identify non-malaria causes of illness in patients with negative malaria tests will facilitate more accurate diagnoses and reduce reliance on clinical judgment.

Another important reason for reliance on clinical findings was their positive experiences with empirical treatments and the undesirable outcomes when treatment was withheld. This non-adherence was more common among senior workers who prioritized personal experience over guidelines. Similarly, reports indicate that physicians with higher levels of training and experience are more likely to base treatment decisions on patient symptoms [15–17,20]. Participants emphasized the importance of not ignoring clinical findings to avoid missed diagnoses and consequent complications when patients are sent home without medication. Guidelines should ensure both a safe management approach and a reduction in unnecessary drug use. Thus, it is crucial to update healthcare workers with evidence supporting the efficacy of withholding medications when not indicated by guidelines [11,13,14] and ensuring adequate follow-up monitoring. Addressing healthcare workers’ concerns about increased workload due to follow-ups and repetitive testing, as well as additional costs for patients, may involve strategies for more efficient testing and subsidizing follow-up tests.

Despite the availability of guidelines in healthcare facilities, the lack of training should be addressed by enhancing training and mentorship opportunities to improve adherence, as highlighted by other studies [12,16,21]. Participants suggested that guidelines should allow flexibility for empirical treatment. Therefore, regularly reviewing and assessing the flexibility of clinical guidelines is essential to balance adherence with practical considerations and current evidence. Changing the attitudes of healthcare workers, especially those senior cadres, towards adherence is crucial and involves integrating empirical knowledge with evidence-based practices [16,21]. Without this, adherence enforcement mechanisms may be undermined, as uncovered in the present study, where laboratory personnel were persuaded by prescribers to alter negative results to positive so patients could receive treatment.

Patients’ lack of trust in laboratory results, particularly when results differed from their expectations due to familiarity with malaria symptoms in endemic areas, led to pressure on healthcare workers to deviate from guidelines or to patient self-treatment, as reported elsewhere [22]. This situation resulted in antimalarial drug stock-outs, misuse, and resource misallocation, as noted by the study participants. Educating patients and communities about adhering to guideline-based clinical decisions may help manage expectations and reduce pressure on healthcare workers to prescribe unnecessary treatments. Quantitative studies show that most caregivers in similar Ethiopian endemic areas adhered well to clinical decisions when follow-up was advised without antimalarial or antibacterial prescriptions, despite some caregivers’ non-adherence [13,14].

The study has several limitations. Social desirability bias may have influenced responses due to face-to-face interviews, though this was mitigated by obtaining participant consent, ensuring privacy and confidentiality, and clearly explaining the study’s purpose. Additionally, the findings are not generalizable to larger populations due to the small, non-random sample size and the inclusion of participants only from public healthcare facilities.

## Conclusion

This study highlighted factors contributing to non-adherence to antimalarial prescribing guidelines, including unreliable microscopy service, mistrust in lab results by healthcare workers and patients, and reliance on clinical judgment considering personal experiences. To improve adherence, it is crucial to ensure consistent lab operations, enhance the quality of malaria test results and maintain effective communication between lab personnel and prescribers. Additionally, implementing training and mentoring programs and promoting evidence-based practices are vital for changing healthcare workers’ attitudes and improving adherence. Ensuring efficient patient follow-up and educating patients on the importance of adhering to healthcare workers’ advice are also essential.
